# Supplementary material for: Identification of Freezing-Responsive microRNAs and Their Targets in Chinese Jujube by Small RNA and Degradome Sequencing
Source: Int J Mol Sci. 2026 Sep 4;27(17):7912. doi: 10.3390/ijms27177912 (PMC13566890; doi:10.3390/ijms27177912)
Supplement: Supplementary file 1 [file ijms-27-07912-s001.zip › Supplementary Material S2/novel_miR_43.pdf]

Provisional ID	:	novel_miR_43
Score total	:	124.3
Score for star read(s)	:	3.9
Score for read counts	:	122.5
Score for mfe	:	0.1
Score for randfold	:	-2.2
Score for cons. seed	:	
Total read count	:	252
Mature read count	:	53
Loop read count	:	185
Star read count	:	14

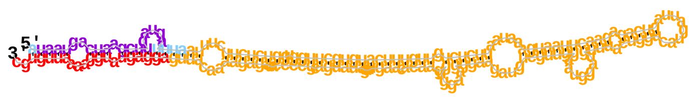

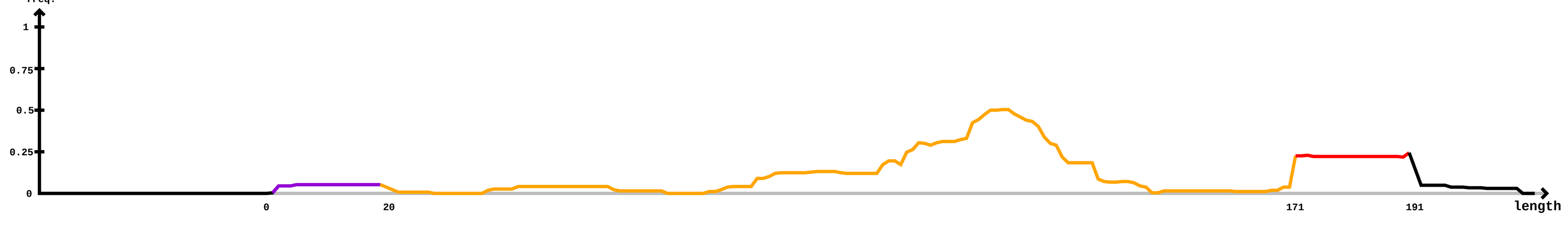

Star

Mature

```
5'- uuucaaaauggaugugaucgaacgagauuauaaguuauaaugacuaagcuugaugauuuaauuucuucucuggugguucguguacuuuguugucucuuaauaaaguaauucaagagacugauuaguacuggucauuuguagguaagguugcuguagaagggaagguggaacacaaguuugauaugacgccccauagagaaaacauugaggaguauggaaaauugugccgugagaggacaaacaagucca -3'   obs
    uuucaaaauggaugugaucgaacgagauuauaaguuauaaugacuaagcuugaugauuuaauuucuucucuggugguucguguacuuuguugucucuuaauaaaguaauucaagagacugauuaguacuggucauuuguagguaagguugcuguagaagggaagguggaacacaaguuugauaugacgccccauagagaaaacauugaggaguauggaaaauugugccgugagaggacaaacaagucca         exp
    .(((((...(((.(((((((......)))))))...((((((..(((.((((.....((((((...((((((((.((((((((((((((((.((((((.....((((((((((.(((((........))))).))))......)))))))....)))))......).)))).))))...)))))).)))))..))))))...))))))))))).)))...)))))))))))))((((......)))).
```

read	reads	mm	sample
auaaugacuaagcuugauga	1	0	LT-3
uaaugacuaagcuugauga	6	0	JT-3
uaaugacuaagcuugauga	5	0	JT-2
ugacuaagcuugaugauuuaauu	2	0	JCK-3
gugguucguCuacuuuguugu	2	1	JCK-3
gugguucguCuacuuuguugu	1	1	JCK-1
gugguucguCuacuuuguugu	2	1	JCK-2
ugguucguguacuuuguuguc	2	0	LT-2
ucguguacuuuguugucucuuaaua	1	0	JT-3
ucguguacuuuguugucucuuaaua	3	0	JT-2
ucaagagacugauuaguacugg	1	0	JCK-3
ucaagagacugauuaguacugg	1	0	JCK-2
ucaagagacugauuaguacuggu	1	0	JCK-1
aagagacugauuaguacuggucauuuguag	2	0	JCK-1
aagagacugauuaguacuggucauuuguag	1	0	LT-3
agagacugauuaguacuggucauuuguag	2	0	JCK-3
agagacugauuaguacuggucauuuguag	2	0	JCK-2
gagacugauuaguacuggucauuuguag	1	0	LT-1
cugauuaguacuggucauuuguag	9	0	LCK-3
cugauuaguacuggucauuuguag	1	0	LT-1
cugauuaguacuggucauuuguag	1	0	JCK-1
cugauuaguacuggucauuuguag	2	0	JCK-2
gauuaguacuggucauuuguag	1	0	JCK-2
gauuaguacuggucauuuguagguaag	2	0	LT-1
auuaguacuggucauuuguag	3	0	LT-3
auuaguacuggucauuuguagguaag	2	0	JCK-2
uuaguacuggucauuuguag	1	0	LT-3
acuggucauuuguagguaa	1	0	JCK-2
cuggucauuuguagguaagg	1	0	LT-1
Aagguaagguugcuguagaagg	3	1	LT-2
Aagguaagguugcuguagaaggg	1	1	JCK-2
Aagguaagguugcuguagaagggaag	6	1	JT-3
Aagguaagguugcuguagaagggaag	1	1	JT-2

Star   Mature

```
uuuucaaaauggaugugaucgaacgagauuauaaguuauaaugacuaagcuugaugauuuaauuucuucucugguggguucguguacuuuguugucucuuaauaaaguaauucaagagacugauuaguacuggucauuuguagguaagguugcuguagaagggaagguggaacacaaguuugauaugacgccccauagagaaaacauugaggaguauggaaaauugugccgugagaggacaaacaagucca
............................................................................................................................................Cagguaagguugcuguagaagggaag.....................................................................................	1	1	JT-3
............................................................................................................................................Aagguaagguugcuguagaagggaaggug..................................................................................	2	1	LT-2
.............................................................................................................................................agguaagguugcuguagaaggga.......................................................................................	3	0	JT-3
.............................................................................................................................................agguaagguugcuguagaaggga.......................................................................................	2	0	JT-2
.............................................................................................................................................agguaagguugcuguagaagggaaggug..................................................................................	1	0	LT-1
...............................................................................................................................................guaagguugcuguagaaggg........................................................................................	2	0	LT-2
...............................................................................................................................................guaagguugcuguagaaggg........................................................................................	2	0	LT-3
...............................................................................................................................................guaagguugcuguagaaggga.......................................................................................	1	0	LT-3
...............................................................................................................................................guaagguugcuguagaagggaa......................................................................................	1	0	JT-2
...............................................................................................................................................guaagguugcuguagaagggaa......................................................................................	1	0	JT-3
...............................................................................................................................................guaagguugcuguagaagggaagg....................................................................................	2	0	JCK-2
...............................................................................................................................................guaagguugcuguagaagggaaggu...................................................................................	2	0	JT-1
...............................................................................................................................................guaagguugcuguagaagggaaggugg.................................................................................	1	0	JT-2
...............................................................................................................................................guaagguugcuguagaagggaaggugg.................................................................................	7	0	JT-3
...............................................................................................................................................guaagguugcuguagaagggaaggugga................................................................................	1	0	LT-2
................................................................................................................................................uaagguugcuguagaagg.........................................................................................	2	0	LT-1
................................................................................................................................................uaagguugcuguagaagg.........................................................................................	2	0	LT-3
................................................................................................................................................uaagguugcuguagaagggaagg....................................................................................	1	0	LCK-3
................................................................................................................................................uaagguugcuguagaagggaagg....................................................................................	1	0	LT-1
................................................................................................................................................uaagguugcuguagaagggaagg....................................................................................	12	0	JT-1
................................................................................................................................................uaagguugcuguagaagggaaggu...................................................................................	1	0	JCK-3
................................................................................................................................................uaagguugcuguagaagggaaggu...................................................................................	1	0	JCK-1
.................................................................................................................................................aagguugcuguagaagggaaggu...................................................................................	1	0	LT-3
.................................................................................................................................................aagguugcuguagaagggaaggu...................................................................................	1	0	LT-2
.................................................................................................................................................aagguugcuguagaagggaaggu...................................................................................	1	0	LT-1
.................................................................................................................................................aagguugcuguagaagggaaggugga................................................................................	1	0	JT-3
..................................................................................................................................................agguugcuguagaagggaagg....................................................................................	1	0	LT-2
..................................................................................................................................................agguugcuguagaagggaaggu...................................................................................	1	0	LT-3
..................................................................................................................................................agguugcuguagaagggaaggugg.................................................................................	6	0	JT-3
..................................................................................................................................................agguugcuguagaagggaaggugg.................................................................................	3	0	JT-2
....................................................................................................................................................guugcuguagaagggaaggu...................................................................................	1	0	JCK-3
.....................................................................................................................................................uugcuguagaagggaaggu...................................................................................	1	0	LCK-3
.....................................................................................................................................................uugcuguagaagggaaggugga................................................................................	4	0	LT-2
......................................................................................................................................................ugcuguagaagggaaggugg.................................................................................	2	0	LT-3
.........................................................................................................................................................uguagaagggaaggugga................................................................................	3	0	LCK-3
..........................................................................................................................................................guagaagggaagguggaacacaa..........................................................................	1	0	LT-2
..........................................................................................................................................................guagaagggaagguggaacacaaguuuga....................................................................	1	0	JCK-1
...........................................................................................................................................................uagaagggaagguggaacaca...........................................................................	24	0	JT-1
...........................................................................................................................................................uagaagggaagguggaacacaaguuuga....................................................................	1	0	LT-2
............................................................................................................................................................agaagggaagguggaacacaaguuug.....................................................................	2	0	LCK-3
............................................................................................................................................................agaagggaagguggaacacaaguuuga....................................................................	3	0	LT-2
.............................................................................................................................................................gaagggaagguggaacaca...........................................................................	2	0	JCK-2
.............................................................................................................................................................gaagggaagguggaacacaa..........................................................................	1	0	LT-1
.............................................................................................................................................................gaagggaagguggaacacaaguuugau...................................................................	1	0	LT-3
.............................................................................................................................................................gaagggaagguggaacacaaguuugaua..................................................................	2	0	JCK-1
.............................................................................................................................................................gaagggaagguggaacacaaguuugaua..................................................................	2	0	JCK-3
..............................................................................................................................................................aagggaagguggaacacaa..........................................................................	2	0	LT-2
..............................................................................................................................................................aagggaagguggaacacaaguuugaua..................................................................	1	0	JT-3
..............................................................................................................................................................aagggaagguggaacacaaguuugaua..................................................................	4	0	JT-2
................................................................................................................................................................gggaagguggaacacaag.........................................................................	1	0	LT-3
....................................................................................................................................................................agguggaacacaaguuugau...................................................................	1	0	LT-3
....................................................................................................................................................................................ugauaugacgccccauaga....................................................	1	0	LT-3
...........................................................................................................................................................................................acgccccauagagaaaacauugagg.......................................	2	0	JCK-2
...........................................................................................................................................................................................acgccccauagagaaaacauugagg.......................................	1	0	JCK-1
.............................................................................................................................................................................................................auugaggaguauggaaaauugu........................	1	0	LT-1
.............................................................................................................................................................................................................auugaggaguauggaaaauugugccgugag................	1	0	LT-3
...............................................................................................................................................................................................................ugaggaguauggaaaauugug.......................	1	0	JCK-3
...............................................................................................................................................................................................................ugaggaguauggaaaauugugc......................	1	0	LT-3
...............................................................................................................................................................................................................ugaggaguauggaaaauugugcc.....................	1	0	LT-1
...............................................................................................................................................................................................................ugaggaguauggaaaauugugccgugag................	2	0	LT-2
.................................................................................................................................................................................................................agAaguauggaaaauugugc......................	1	1	LCK-1
.................................................................................................................................................................................................................aggaguauggaaaauugugc......................	27	0	LCK-2
.................................................................................................................................................................................................................aggaguauggaaaauugugc......................	21	0	LCK-1
.................................................................................................................................................................................................................Gggaguauggaaaauugugc......................	1	1	LCK-1
...................................................................................................................................................................................................................gaguauggaaaauugugccgugagaggaca..........	1	0	JT-1
....................................................................................................................................................................................................................aguauggaaaauugugccgugagagg.............	1	0	JCK-3
....................................................................................................................................................................................................................................ccgugagaggacaaacaag....	5	0	JT-2
....................................................................................................................................................................................................................................ccgugagaggacaaacaag....	3	0	JT-3
```
